# Exploring the potential of cardiopulmonary exercise testing (CPET) for tailored pulmonary rehabilitation in people with interstitial lung disease (ILD): A systematic review protocol

**DOI:** 10.3310/nihropenres.13706.2

**Published:** 2025-07-04

**Authors:** Ben Bowhay, Craig A Williams, Michael A Gibbons, Chris J Scotton, Owen W Tomlinson

**Affiliations:** 1 Clinical & Biomedical Sciences, University of Exeter, Exeter, England, UK; 2 Academic Department of Respiratory Medicine, Royal Devon University Healthcare NHS Foundation Trust, Exeter, England, UK; 3 NIHR Biomedical Research Centre, University of Exeter, Exeter, England, UK; 4 Public Health & Sport Sciences, University of Exeter, Exeter, England, UK

**Keywords:** Exercise testing, exercise training, respiratory disease, review

## Abstract

**Background:**

This review aims to identify which cardiopulmonary exercise test (CPET) derived variables can be used to personalise pulmonary rehabilitation (PR) for people with interstitial lung diseases. A ‘one size fits all’ approach does not benefit every patient due to a multitude of unique characteristics, subsets and phenotypes. No ILD-specific, tailored pulmonary rehabilitation guidelines exist in this area and exercise programme development is lacking. This leads to wide variation in the success within the literature and clinical practice.

**Methods:**

MEDLINE, Embase, CINAHL, SPORTDiscus and the Cochrane Database of Systematic Reviews will be searched to identify studies that utilise CPET variables for PR development. Quality assessment is to be performed using the Critical Appraisal Skills Program (CASP) checklists for single cohort studies and randomised controlled studies.

**Discussion:**

The primary outcomes found within the included studies for peak volume of oxygen consumption (VO _2peak_), work rate (WR _peak_), oxygen consumption at anaerobic threshold (VO _2_-AT), heart rate and rate of perceived exertion (RPE) would help determine which variables are optimal for prescription success. Identification of reliable methods to tailor PR for people with interstitial lung disease would enhance what is already known and potentially lead to best practice guideline development.

**Registration:**

In accordance with the guidelines, this systematic review protocol was registered with the International Prospective Register of Systematic Reviews (PROSPERO) on 07 May 2024 (registration number CRD42024543174).

## Plain Language Summary

Interstitial lung diseases (ILD) are a group of around 200 long-term lung conditions, which often lead to lung failure. Pulmonary Rehabilitation (PR) is a mix of exercise, advice and education that is used to support people with ILD (pwILD) to help maintain their lung function, general function, and quality of life.

However, a ‘one size fits all’ PR approach may not be best, due to the many challenges found for ILD as a condition. Therefore, using a gold-standard tool like Cardiopulmonary Exercise Testing (CPET) can help to assess these challenges; as we look to understand how the heart, lungs and muscle work during exercise for each pwILD and these outcomes would help support person-centred exercise selection.

The systematic review aims to discover which CPET outcomes can be used to tailor PR for people with ILD. A range of databases (MEDLINE, Embase, CINAHL, SPORTDiscus and the Cochrane Database of Systematic Reviews) will be searched to identify studies that meet this aim, and checklists (Critical Appraisal Skills Program, CASP) will then be used to rate the quality of the included studies.

No tailored PR guidelines are available for pwILD, which leads to many differences in the success of PR. We will review the current evidence, with the aim to see which CPET outcomes can be used for PR selection. The systematic review results would help to show if technically appropriate methods can be developed for tailored PR in pwILD and support the design of future studies that can guide PR practice.

## Introduction

### Rationale

Interstitial lung disease (ILD) is a heterogeneous group of approximately 200 chronic lung conditions, which are associated with lung parenchymal fibrosis and/or interstitial inflammation ^1^. Consequently, respiratory failure is the primary cause of mortality in people with ILD ^2^. According to 2017 figures, the median age-standardised incidence rates (ASIR) of ILD in United Kingdom (UK) men was 10.92 per 100,000, and 6.7 per 100,000 for UK women ^3^. From the period between 2001 and 2017, the ASIR has risen by 21.27% for UK men and 25.39% for UK women ^3^. In addition, the median age-standardised death rates indicated that ILD related mortality was 2.04 (IQR 1.13–2.71) per 100 000 population for men and 1.02 (0.68–1.37) per 100,000 population for women ^3^. Unfortunately, ILD are chronic conditions that cannot be cured, therefore maintaining lung function, general function, and quality of life (QoL) of people with ILD (pwILD) is a key focus for patients and clinicians alike ^4^.

A joint statement produced by the American Thoracic Society (ATS) and European Respiratory Society (ERS) recommends regular exercise training and pulmonary rehabilitation (PR) to enhance cardiorespiratory health ^5^, whereby exercise is acknowledged as a planned, structured, and repetitive form of physical activity which is performed for the improvement or maintenance of physical fitness ^6^. International guidelines highlight that exercise training is regarded as a cornerstone of PR ^5^. However, individual patient responses to exercise training are highly variable and a ‘one size fits all’ training approach does not benefit every pwILD ^7^, thus placing the onus on to effective PR design.

Pulmonary rehabilitation is an intervention which is developed following comprehensive diagnostic evaluation by a multidisciplinary team; the aim is to utilise these assessment outcomes to establish a patient-centred therapeutic program which consists of exercise and patient education to support behaviour change, but also improve physical and mental health for people with chronic respiratory diseases ^8^. The British Thoracic Society (BTS) explicitly recommends that PR should be offered to symptomatic individuals with chronic respiratory disease, including pwILD ^8^. Therefore, National Health Service in the United Kingdom, alongside the National Respiratory Audit Programme (NRAP) are aiming to drive forward the quality of PR services ^9^.

A 2021 Cochrane review of PR in pwILD has found “moderate-certainty” evidence associated with enhanced functional exercise capacity (measured by the six-minute walk test, 6MWT) and “low-certainty” evidence that suggests that PR in pwILD may improve maximum exercise capacity (WR _peak_) and dyspnoea ^10^. These benefits are postulated to be sustained in the longer term (e.g. 12 months duration) and there is also no evidence that adverse events were found in the pwILD ^11^. Therefore, PR is not only useful for enhancing QoL in pwILD, but functional capacity as well ^11^. However, no PR guidelines have been developed specifically for pwILD at present, despite them being available for other chronic respiratory diseases such as COPD ^12^. Therefore, the James Lind Alliance has made ‘best exercise program for people with Pulmonary Fibrosis’ part of their top ten research priorities ^13^.

The main challenge for development of PR guidelines in ILD is related to the breath of pathophysiological impairments that occur across the different ILD subgroups, which may lead to variation in exercise tolerance and capacity ^14^. Restrictions in exercise tolerance may be due to ventilation/perfusion (V/Q) mismatch, which occurs when either airflow or blood flow in the lungs is impaired and abnormal ventilatory mechanics can occur at different points for pwILD ^14^. Therefore, pwILD may often require more ventilatory load and capacity to sustain exercise, whilst also attempting to combat muscle fatigue, commonly because of poor blood oxygenation ^14^; this may lower ATP, increase lactate and cause a rise in VCO _2_^11^. Gas exchange insufficiency, central haemodynamic impairment and muscle deconditioning are prevalent in the ILD population ^14^. Therefore a tailored approach to exercise prescription may provide enhanced physical function outcomes, as tailoring could account for these mechanisms of exercise intolerance in chronic lung diseases ^15^. However, due to the complex characteristics of ILD, a review of the methods used for tailored exercise prescription is required, before comprehensive guidelines in this population can be developed.

The BTS Clinical Statement on pulmonary rehabilitation states that a validated exercise test should be conducted as a core component of PR programmes to inform tailored prescription ^8^. Within ILD management, field tests such as 6-minute walk distance (6MWD) ^16– 18^ and the incremental shuttle walk test (ISWT) ^19^ are often used as a functional outcome because they are valid, easy to implement and cost-effective ^16– 19^. However, field tests cannot highlight the full spectrum of pathophysiological mechanisms which limit exercise tolerance, and safety issues such as ischemia or arrhythmias cannot be easily detected ^20^. Both aspects are of upmost importance when looking to prescribe exercise in the ILD population, due to the aforementioned physiological impairments ^14^. Therefore, the focus of this systematic review is to explore the potential utility of CPET to provide the precision required for tailored exercise prescription in ILD.

Tailored exercise via CPET has been earmarked as the future of PR, whereby a comprehensive evaluation of pathophysiological systems serves to indicate responses to exercise, and therefore these values could support effective PR programming ^14^. Evidence suggests that CPET is now the gold standard for the causal evaluation of exercise intolerance in patients with long term pulmonary conditions ^21^. It has been demonstrated that CPET is a safe and valuable method for the comprehensive evaluation of cardiac, pulmonary and muscle function ^21^, whilst also recognising physiological factors limiting exercise such as dyspnoea ^22^.

Exercise prescription methods which are tailored to the cardiovascular, pulmonary, and peripheral muscle metabolic limitations of the individual patient have the potential to be the cornerstone of tailored PR ^14^. Recent evidence suggests that tailored, moderate-intensity continuous aerobic exercise (at 60% maximum heart rate; HR _max_) and high-intensity interval training (at 80% HR _max_) is beneficial in pwILD for improving WR _peak_ and dysponea ^23^. Moreover, tailored exercise training can enhance antioxidant buffering capacity in people with idiopathic pulmonary fibrosis (IPF), which may corelate with enhanced muscle fatigue resilience ^24^. This clearly demonstrates the functional and physiological value PR can have when using tailored approaches.

However, for widespread success of tailored PR programmes in ILD, implementation of exercise prescription principles (e.g. specificity, frequency, intensity, timing, type, overload, progression, adaptation, and reversibility) should be followed and reported accordingly ^5^. At present, it is not clear if this is currently done for pwILD; the current systematic review helps provide an overview of practice regarding the components of exercise prescription in this population.

There is also a need to develop a valid, reliable, and consistent method for personalising PR based on CPET outcome measures. Several metrics could be used (e.g. VO _2peak_ or heart rate), but it is unclear what metrics are currently used and how they are implemented. This systematic review will seek to address this, with the intention of highlighting the CPET-derived exercise prescription variables that have been used to tailor PR for pwILD.

### Objectives

This review aims to identify which cardiorespiratory variables, and intensities, derived from CPET can be used to personalise PR for pwILD. 

## Methods

### Eligibility criteria

The PRISMA-P has been used to guide this systematic review protocol ^25^. Eligibility for inclusion in this review include the following elements of the PICOS framework ( Table 1); conference abstracts will be excluded; articles which are not published in English, unless an English translation is available, will be excluded; there will be no restriction on publication date, or location.

**Table 1.** Inclusion and exclusion criteria.

Selection	Inclusion criteria	Exclusion criteria
Population(s)	ILD patients of all ages, genders and disease severities	Non-ILD patients
Intervention(s)	Tailored pulmonary rehabilitation or exercise programmes, which have been personalised using CPET derived values.	Articles which only describe ‘physical activity’ interventions and not ‘exercise training’ or ‘pulmonary rehabilitation’ will be excluded. Single bouts of exercise would also not classify as exercise training in this context.
Comparators	Control where possible	Not specified
Outcomes	Peak volume of oxygen consumption (VO _2peak_); Peak work rate (WR _peak_); Volume of oxygen consumption at anaerobic threshold (VO _2_-AT); Heart Rate (BPM); Rate of perceived exertion (RPE)	Outcomes which do not relate to inclusion criteria.
Study design	Full text studies only. Published in English. No restriction on publication date, or location.	Non-full text articles and conference abstracts will be excluded. Articles which are not published in English, unless an English translation is available, will be excluded.
Setting	All settings	Nil

***Population***

People of all ages, sexes and ethnic groups that have been diagnosed with any ILD subtype.

***Intervention***

Tailored PR or exercise programmes, which have used CPET derived values. Articles that only describe ‘physical activity’ interventions, and not ‘exercise training’ or ‘pulmonary rehabilitation’, will be excluded. Single or acute bouts of exercise will not classify as exercise training.

***Comparison***

Outcomes of PR regimens against group baselines, and where possible, against control groups as well will be compared.

***Outcomes***

Outcomes will be derived from CPET values. It is anticipated this may include, but will not be limited to:

a) Peak volume of oxygen consumption (VO _2peak_), the gold-standard measure of cardiorespiratory fitness, expressed as mL.kg ^-1^.min ^-1^ or L.min ^-1^;
b) Peak work rate (WR _peak_), expressed in watts (W), the maximum achieved work rate during an incremental CPET;
c) Volume of oxygen consumption at anaerobic threshold (VO _2_-AT) expressed as mL.kg ^-1^.min ^-1^, L.min ^-1^, or %VO _2peak_;
d) Rate of perceived exertion (RPE) that can be measured on a 0–10 RPE scale or a Borg 6–20 scale.
e) Rate of perceived exertion (RPE) that can be measured on a 0–10 RPE scale or a Borg 6–20 scale.

***Study designs***

All quantitative study designs will be included; for instance, RCT’s, single cohort studies and case studies. Only full-text, original articles will be included.

### Information sources

Electronic searches will be conducted using Ovid® (Ovid Technologies Inc., New York, NY, USA), incorporating Ovid MEDLINE® (US National Library of Medicine, Bethesda, MD, USA), Ovid EmbaseTM (Elsevier Inc., Philadelphia, PA, USA), EBSCO databases CINAHL Ultimate and SPORTDiscus (EBSCO Information Services, MA, USA) plus CENTRAL and the Cochrane Library (John Wiley and Sons Ltd, Hoboken, NJ, USA) will all be searched up to January 2025.

### Search strategy

The search strategy is available at OSFHOME: Exploring the potential of cardiopulmonary exercise testing (CPET) for individualised pulmonary rehabilitation in people with interstitial lung disease (ILD): A systematic review protocol: Search_Strategy.docx. Available at: https://doi.org/10.17605/OSF.IO/43N29.

Following completion of the Ovid MEDLINE® strategy, specific syntax and subject headings will be created for all Ovid, EBSCO and Cochrane database searches.

## Study records

### Data management

Independent data extraction will take place using data extraction forms developed for the study. Data to be extracted and included in the table of ‘overview of included studies’ will include author and year, purpose of study, setting, country, sample size, participant demographic and clinical diagnosis, treatment types, study design, primary outcome measures, losses and exclusion of participants. EndNote© 20 ^26^ (Clarivate, London, UK) will be utilised for reference storage.

### Selection process

A screen of the initial search results will be performed by title and abstract for eligibility and inclusion. The search results will be checked by a single reviewer (BB), then an experienced second reviewer (TBC) will perform a check of 10%, then a third reviewer (TBC) will check for differences and achieve a consensus through discussion with the reviewers.

### Data collection process

Data will be collected by a single reviewer (BB) using a data extraction form. A random double data extraction (10%) check will then be performed by a second reviewer (TBC). Any differences will be highlighted by a third reviewer (TBC), then a discussion with the reviewers will be performed to achieve a consensus. Any missing data will be requested from the study authors, before removal from the review, if no reply is provided.

### Data items

The PICOS inclusion and exclusion criteria ( Table 1) will be used by (BB and TBC) to as a framework to collate and review all the full text articles which are potentially relevant for the review. If disagreement occurs between the reviewers, then a discussion with the third reviewer (TBC) will be conducted to reach consensus. Outcomes will be derived from CPET values. We anticipate that the main outcomes will be displayed in a summary of findings table using GRADEpro ^27^.

### Risk of bias in individual studies

Quality assessment of the included studies will be performed by two reviewers (BB; TBC) using the Critical Appraisal Skills Program (CASP) checklists for single cohort studies ^28^ and randomised controlled studies ^29^, thus accounting for the anticipated variances in study designs for this review. A third reviewer (TBC) would then confirm the results and lead the discussion towards consensus of the included studies. The CASP checklist items are to be marked “Y” (Yes) if well described, “N” (No) if inadequate, and unclear items with a “U” (Unclear).

***Reporting quality***

The Consensus on Exercise Reporting Template (CERT) assessment form will be used to check the reporting of exercise programme domains ^30^. This would support researchers to develop effective exercise interventions, that are well constructed and reproduceable. Using the CERT would also help policymakers with making exercise recommendations and guide peer reviewers in manuscripts evaluation ^30^.

## Data

### Synthesis

All analyses will be performed using Review Manager ^31^.

a. Data pertaining to variables used to prescribe, and how, will be narratively discussed and analysed using frequency statistics.
b. Where possible, analyses of between groups to receive tailored PR against controls will be undertaken. If no mean change and standard deviation of change are reported, then a corresponding author data request will be made. If no data or response is gained, then results will be calculated using methods in the Cochrane Handbook ^32^. The summary effect size is estimated by using mean difference (MD) with 95% CI for continuous outcomes.
Standardized mean differences (SMD) will be utilised instead, if different methods or scales are used for the outcome. Heterogeneity will be estimated from the MD and SMD via a χ ^2^ test. In addition, the I ^2^ test will also be utilised to add extra analysis. Signifficant heterogeneity will be reached at P<0.1 in the χ ^2^ test and >50% in the I ^2^ test. A random-effect or fixed-effect model will be selected to merge the outcomes.

***Meta-bias(es)***

Reporting bias would be explored by examining if the protocol was published prior to study participant recruitment. A Clinical Trial Register at the International Clinical Trials Registry Platform of the World Health Organisation screen would also be conducted. Selective outcome reporting bias would be evaluated by comparing the fixed effect estimate against the random effects model to examine the potential for sample bias. If this occurs, then a random effects estimate would be advantageous, when compared against a fixed effect estimate. Reporting bias could also be examined by funnel plots if ≥10 studies are identified.

### Confidence in cumulative evidence

The GRADE tool ^33^ would be used to assess the evidence for the primary outcome of CPET values. Analyses and presentation of the results would be stated in a 'Summary of findings' table, generated using GRADEpro GDT ^27^ software.

## Discussion

The current systematic review has been developed to explore how CPET-derived outcomes can help support tailored PR design for pwILD. At present, there are no systematic reviews that provide insights into how CPET values can be utilised for tailored exercise and PR programming in pwILD.

The most relevant databases have been selected specifically for this systematic review, and in addition, all study designs have been included, which although a strength of this systematic review, may add complexity when looking to interpret the results across the board. Thus, due to the potential variability in both the condition subgroups within ILD and how PR interventions may be designed, it is possible that separate analyses may be required.

A potential limitation of this study would be the focus on CPET, without direct comparison against field tests. The potential strength of this review is that exploring CPET, as a tool for tailoring exercise, may help to indicate beneficial methods for PR prescription in the ILD population. The overall aim following this review is to develop a valid, reliable, and consistent method for tailoring PR based on CPET outcome measures to address the current evidence gap. Several metrics may be used (e.g. VO _2peak_, heart rate), but it is unclear how and why they are utilised in clinical practice for pwILD; therefore, this systematic review aims to fill this research gap.

## Subject index terms

Interstitial Lung Diseases; Pulmonary Rehabilitation; Exercise; CPET; Treatment Outcomes; Training; Physiotherapy; Systematic Review.

## List of abbreviations

CPET: Cardiopulmonary Exercise Testing

ILD: Interstitial Lung disease

UK: United Kingdom

PR: Pulmonary Rehabilitation

VO _2peak_: Peak volume of oxygen consumption

WR _peak_: Peak work rate

VO _2_-AT: Volume of oxygen consumption at anaerobic threshold

RER: Respiratory exchange ratio

V _E_: Minute ventilation

AT: Anaerobic Threshold

HR: Heart Rate

RPE: Rating of perceived exertion

ATS: American Thoracic Society

ERS: European Respiratory Society

BTS: British Thoracic Society

pwILD: people with interstitial lung disease

CASP: Critical Appraisal Skills Program

GRADE: Grading of Recommendations, Assessment, Development, and Evaluations

HRR: Heart Rate Reserve

COPD: Chronic Obstructive Pulmonary Disease

HFA-PEFF: The Heart Failure Association (HFA)-PEFF score

6MWT: Six-minute walk test

6MWD: Six-minute walk distance

ICT: Incremental cycle test

RCT: Randomised controlled trial

## Amendments

In the event of protocol amendments, the date of each amendment will be accompanied by a description of the change and the rationale.

## Ethics approval and consent to participate

Not applicable

## Consent for publication

Not applicable

## Data Availability

No data are associated with this article. Reporting guidelines are available at OSFHOME: Exploring the potential of cardiopulmonary exercise testing (CPET) for individualised pulmonary rehabilitation in people with interstitial lung disease (ILD): A systematic review protocol: PRISMA-P.doc.x. Available at: https://doi.org/10.17605/OSF.IO/43N29 ^25^. Data is available under Creative Commons Zero (CC0): *1.0 Universal*. OSFHOME: Exploring the potential of cardiopulmonary exercise testing (CPET) for individualised pulmonary rehabilitation in people with interstitial lung disease (ILD): A systematic review protocol Available at: https://doi.org/10.17605/OSF.IO/43N29 ^25^ This project contains: Inclusion and exclusion criteria - Protocol.docx Search_Strategy.docx Data is available under Creative Commons Zero (CC0): *1.0 Universal.*
