# Supplementary material for: Exploring the potential of cardiopulmonary exercise testing (CPET) for tailored pulmonary rehabilitation in people with interstitial lung disease (ILD): A systematic review protocol
Source: NIHR Open Res. 2025 Jul 4;4:51. Originally published 2024 Sep 17. [Version 2] doi: 10.3310/nihropenres.13706.2 (PMC12368488; doi:10.3310/nihropenres.13706.2)
Supplement: Peer review history

# Reviewer report: Reviewer response for version 2

**DOI:** 10.3310/nihropenres.15241.r36274

**Published:** 2025-09-03

**Authors:** Winceslaus Katagira

**Affiliations:** 1 Makerere University Lung Institute, Kampala, Uganda

This is a highly relevant systematic review.

 The burden of ILD is increasing globally and poses a growing clinical challenge. Although PR is recognised as a cornerstone of care for chronic lung diseases, there are no ILD-specific, evidence-based guidelines.

 CPET is considered the gold standard for assessing exercise capacity; however, there's a paucity of data on how CPET can be used to guide personalised PR in ILD.

 This protocol addresses that gap.

 Overall, the protocol is well-written, with robust methodology and is clinically significant. It has been registered in PROSPERO. Patient and Public Involvement is commendable. 

 Limitations:

 1. A good number of technologically advanced PR centers are in Asia, where English is not a primary language. Excluding non-English articles may introduce language bias, but the lack of translators is a legitimate limitation/challenge.

 2. Many PR offering centers have limited CPET access; therefore real-world applicability of CPET may be limited.

 3. Limited generalisability to resource-limited settings. CPET is resource-intensive, and most low-resource settings will lack access to CPET.

 4. CPET offers precision in identifying mechanisms of exercise tolerance and captures the physiological responses; however, for many ILD patients, the improvements may be more psychological and behavioural.

 5. The lack of direct comparisons with field tests like ISWT and 6MWT may limit clinic transferability. Perhaps future studies can aim to address this.

Is the study design appropriate for the research question?

Yes

Is the rationale for, and objectives of, the study clearly described?

Yes

Are sufficient details of the methods provided to allow replication by others?

Yes

Are the datasets clearly presented in a useable and accessible format?

Yes

Reviewer Expertise:

Pulmonary Rehabilitation, Post-TB lung disease, Chronic respiratory diseases

I confirm that I have read this submission and believe that I have an appropriate level of expertise to confirm that it is of an acceptable scientific standard, however I have significant reservations, as outlined above.

# Reviewer report: Reviewer response for version 2

**DOI:** 10.3310/nihropenres.15241.r36219

**Published:** 2025-08-20

**Authors:** Samantha Harrison

**Affiliations:** 1 Teesside University,, Middlesbrough, UK

I have no further comments to make

Is the study design appropriate for the research question?

Yes

Is the rationale for, and objectives of, the study clearly described?

Partly

Are sufficient details of the methods provided to allow replication by others?

Yes

Are the datasets clearly presented in a useable and accessible format?

Not applicable

Reviewer Expertise:

Pulmonary Rehabilitation; mixed-methods including codesign and PPIE

I confirm that I have read this submission and believe that I have an appropriate level of expertise to confirm that it is of an acceptable scientific standard.

# Reviewer report: Reviewer response for version 1

**DOI:** 10.3310/nihropenres.14882.r33312

**Published:** 2024-12-16

**Authors:** Samantha Harrison

**Affiliations:** 1 Teesside University,, Middlesbrough, UK

The manuscript reports a systematic review protocol to identify which variables derived from undertaking a CPET are best used to personalise exercise prescription in pulmonary rehabilitation.

 Overall, this is a well presented manuscript and the proposed review should yield interesting results. However, I do wonder about the clinical applicability of findings as CPET testing is seldom used by pulmonary rehabilitation providers.

 The terms: tailored, individualized and personalized are used inter-changeably. I suggest selecting one terms and applying consistently throughout.

 The authors state that individual responses to exercise training vary - is this based on research or clinical experience?  I think it needs to be acknowledged that this may in part be due to variation in physiological response but psychological and behavioural factors are also likely to be important.

 The authors state that personalized PR guidelines are available for other chronic respiratory diseases such as COPD yet reference 12 is a review and not guidelines. Furthermore, this published review  provides information on personalised exercise prescription for patients with CRD, including ILD. Please explain how the systematic review proposed builds on this one conducted in 2019 (Armstrong).

 Although the protocol is generally well written, at times the writing style could be more clear and concise e.g. “thus placing the onus onto effective PR design” = emphasizing the importance of individualized PR (this point is repeated). These benefits are postulated to be sustained in the longer term” = benefits are sustained long-term (add duration ?12m).

 Please remove detail about methods (searches) from the objective statement.

 I think the objective could be clearer – I suggest clarifying what is meant by personalized

 . Do the authors mean variables useful to prescribe exercise intensity in this group?

 Did PPIE members feed into the protocol development?

 Why will the searches only be up until January 2024. Is the review already underway and if so is it close to publication otherwise searches will be 12m out of date and should be updated.

 There is some repetition in the methods section. For example, as written the data management section and data collection process could be merged. The outcomes of interest are stated twice.

Is the study design appropriate for the research question?

Yes

Is the rationale for, and objectives of, the study clearly described?

Partly

Are sufficient details of the methods provided to allow replication by others?

Yes

Are the datasets clearly presented in a useable and accessible format?

Not applicable

Reviewer Expertise:

Pulmonary Rehabilitation; mixed-methods including codesign and PPIE

I confirm that I have read this submission and believe that I have an appropriate level of expertise to confirm that it is of an acceptable scientific standard, however I have significant reservations, as outlined above.

# Reviewer report: Reviewer response for version 1

**DOI:** 10.3310/nihropenres.14882.r32957

**Published:** 2024-10-15

**Authors:** Enya Daynes

**Affiliations:** 1 NIHR Leicester Biomedical Research Centre- Respiratory, University Hospitals of Leicester NHS Trust (Ringgold ID: 4490), Leicester, England, UK; 2 University of Leicester Department of Respiratory Sciences (Ringgold ID: 574216), Leicester, England, UK

Thomlinson and colleagues report a protocol for a systematic review to explore the potential for individualising Pulmonary Rehabilitation (PR) in those with Interstitial Lung Disease (ILD), or more specifically, individualizing the aerobic exercise component of PR. They make a strong case of need for this work, particularly highlighting the lack of guidance specific to those with ILD, which is compounded but the challenges that a large proportion of literature is not focused towards these patients. Historically, people with ILD were not always included in PR programmes, but it was recommended in the 2023 BTS clinical statement to include those with ILD in PR, which is now captured in national audits (i.e. National Respiratory Audit Programme) [Ref 1] , ( https://www.nrap.org.uk/) . Therefore, this is clearly an important area of work and aligns with priorities for research.

 The methods described are appropriate and robust, following standardised systematic review frameworks and where possible performing meta-analyses. It may be necessary to include sub-group analyses as the authors highlight, and particular considerations in regard to duration of programmes, dose of exercise prescription, disease characteristics (including sub-groups of ILD) and setting of delivery could be valuable. Additionally, the authors intend to include studies from outside of the UK and therefore adjusting for the variability of PR delivery (inpatient vs outpatient, duration of programme, etc) may be necessary in understanding and interpreting the results.

 There are several strengths to this work. The authors present a robust systematic review plan in an area that is pertinent. They plan to include a variety of studies, and to contact authors for further information/data. They have scheduled bias analysis and are open to appropriate sub-group analysis. Though this work will not be without its challenges. Defining PR will be crucial to the success of this study, which is in part answerable to the requirement of including an exercise

 intervention, but the definition can vary globally [Ref 2] . Reporting of interventions, particularly where there is a degree of personalisation or tailoring is often poor or difficult to interpret, and similarly, the reporting and combining of results may be challenging {Ref 4] .

 Finally other considerations that may be useful is determining or defining personalisation. There is a degree of personalisation in PR programmes, typically prescribed by the Incremental Shuttle Walking Test or 6 Minute Walk Test (the latter highlighted by authors), and despite personalisation/tailoring predominantly influencing the duration and absolute intensity of the aerobic exercise component, this would still be considered personalisation. Perhaps personalisation of PR exists on a spectrum from minimal adjustments of the same programme to a completely individualized exercise programme. Somewhere in between lays the authors ambitions of personalising the type, time and intensity of aerobic exercise within PR for those with ILD.

 This is a well written protocol and clearly articulated the needs of the research. I would recommend minor corrections to firstly consider the Incremental Shuttle Walking Test in the introduction and its impact on personalisation, particularly as its use continues to grow within PR services, and to revise the references, notably to include the James Lind Alliance top 10 priorities for Pulmonary Rehabilitation as the current reference is incorrect.

 This is an important area of research on a long-neglected group of patients which has the potential to have significant impact to the literature, patients and healthcare providers. As the authors highlight, in some areas Cardiopulmonary Exercise Testing (CPET) is becoming increasingly accessed, however this is not the case for all services, particularly with limited access to equipment and resources. This is the first step in understanding personalisation through CPET in those with ILD, however further research could continue to explore the translation of this concept into other, lower resourced areas of care delivery.

Is the study design appropriate for the research question?

Yes

Is the rationale for, and objectives of, the study clearly described?

Yes

Are sufficient details of the methods provided to allow replication by others?

Yes

Are the datasets clearly presented in a useable and accessible format?

Not applicable

Reviewer Expertise:

Senior Specialist Physiotherapist in Pulmonary Rehabilitation, expertise in Pulmonary Rehabilitation, Breathlessness Management, Systematic Reviews, Quantitative Methods, Qualitative Methods.

I confirm that I have read this submission and believe that I have an appropriate level of expertise to confirm that it is of an acceptable scientific standard.

## References

[1] : British Thoracic Society Clinical Statement on pulmonary rehabilitation. *Thorax* .2023;78(Suppl 5) : 10.1136/thorax-2023-220439 s2-s15 10.1136/thorax-2023-220439 37770084

[2] : Defining Modern Pulmonary Rehabilitation. An Official American Thoracic Society Workshop Report. *Annals of the American Thoracic Society* .2021;18(5) : 10.1513/AnnalsATS.202102-146ST e12-e29 10.1513/AnnalsATS.202102-146ST PMC808653233929307

[3] : A new framework for developing and evaluating complex interventions: update of Medical Research Council guidance. *BMJ* .2021;374: 10.1136/bmj.n2061 n2061 10.1136/bmj.n2061 34593508 PMC8482308
